# C-Reactive Protein to Albumin Ratio is Associated with Disease Activity in Anti-Neutrophil Cytoplasmic Antibody Associated Vasculitis

**DOI:** 10.31138/mjr.34.1.71

**Published:** 2023-03-31

**Authors:** Dilek Barutcu Atas, Gizem Kumru Sahin, Şule Şengül, Bülent Kaya, Saime Paydaş, Fatma Alibaz-Oner, Haner Direskeneli, Serhan Tuglular, Ebru Asicioglu

**Affiliations:** 1 Marmara University, School of Medicine, Department of Internal Medicine, Division of Nephrology, Istanbul, Turkey,; 2 Ankara University, School of Medicine, Department of Internal Medicine, Division of Nephrology, Ankara, Turkey,; 3 Çukurova University, School of Medicine, Department of Internal Medicine, Division of Nephrology, Adana, Turkey,; 4 Marmara University, School of Medicine, Department of Internal Medicine, Division of Rheumatology, Istanbul, Turkey

**Keywords:** ANCA-associated vasculitis, Birmingham vasculitis activity score, C-reactive protein to albumin ratio, vasculitis damage index

## Abstract

**Objective/Aim:**

C-reactive protein to albumin ratio (CAR) has recently been recognized as an independent prognostic marker for vasculitides. This study aims to investigate CAR and its relationship with disease activity and damage in prevalent ANCA associated vasculitis (AAV) patients.

**Methods:**

Fifty-one patients with AAV and 42 age-sex-matched healthy controls were enrolled in this cross-sectional study. Birmingham vasculitis score (BVAS) was used to assess vasculitis activity and vasculitis damage index (VDI) to provide information on disease damage.

**Results:**

The median (25^th^–75^th^) age of the patients were 55 (48–61) years. CAR was significantly higher in AAV patients than controls (1.9±2.7 vs 0.7±0.4; p=0.006). The 75^th^ percentile of BVAS was defined as high BVAS (BVAS≥5) and ROC curve analysis showed that CAR≥0.98 predicted BVAS≥5 with 70.0% sensitivity and 68.0% specificity (AUC:0.660, CI: 0.482–0.837, p=0.049). When patients with CAR≥0.98 were compared to those without, BVAS [5.0 (3.5–8.0) vs. 2.0 (0–3.25), p<0.001], BVAS≥5 [16 (64.0%) vs 4 (15.4%) patients, p:0.001], VDI [4.0 (2.0–4.0) vs. 2.0 (1.0–3.0), p=0.006], and CAR [1.32 (1.07–3.78) vs. 0.75 (0.60–0.83), p<0.001] were higher whereas albumin [3.8 (3.1–4.3) vs. 4.1 (3.9–4.4) g/dL, p=0.025] and haemoglobin [12.1 (10.4–13.4) vs. 13.0 (12.5–14.2) g/dL, p=0.008] were lower. Multivariate analysis revealed that BVAS [OR(95% CI):1.313 (1.003–1.719), p=0.047] was an independent factor associated with CAR≥0.98 in patients with AAV. Furthermore, correlation analysis showed that CAR significantly correlated with BVAS (r: 0.466, p=0.001).

**Conclusion:**

In this study, we observed that CAR was significantly associated with disease activity in AAV patients and can be used to monitor disease activity.

## KEY MESSAGES:

1. C-reactive protein to albumin ratio (CAR), a composite indicator of inflammation and nutritional status, has recently been recognized as an independent prognostic marker in malignancy, Crohn’s disease, critically ill patients and vasculitides. In this study, we aimed to investigate CAR and its relationship with disease activity and damage in prevalent ANCA vasculitis patients.
2. Inflammation markers are essential for predicting disease related morbidity and mortality in AAV patients. In the present study, we showed that CAR is associated with high disease activity in AAV patients.
3. Currently the cut-off serum CAR level to predict disease activity in AAV patients is unknown. In our study, patients with CAR ≥0.98 had higher BVAS.
4. The VDI was developed as a tool to distinguish chronic damage from active inflammation or persistent disease. In our study median VDI was 4 and was significantly higher in patients with CAR ≥0.98.
5. Close monitoring, early recognition and timely treatment of disease activity may limit patient morbidity and mortality.

## INTRODUCTION

Anti-neutrophil cytoplasmic antibody-associated vasculitis (AAV) is characterized by inflammation and necrosis of small/medium sized blood vessels and the presence of circulating anti-neutrophil cytoplasmic antibody (ANCA). They can present with a wide variety of signs and symptoms and include granulomatous polyangiitis (GPA), microscopic polyangiitis (MPA) and eosinophilic granulomatous polyangiitis.

High plasma C-reactive protein (CRP) levels are frequently observed in AAV patients as well as other chronic systemic inflammatory diseases and are associated with inflammation, oxidative stress and endothelial dysfunction.^1^ Albumin is a negative acute-phase protein produced in the liver. A low level of serum albumin is a poor prognostic factor in AAV and correlates with inflammation and severe kidney injury. It has been shown that AAV patients with nephrotic hypoalbuminemia have a higher incidence of infection, end stage renal disease and all-cause mortality than patients with mild hypoalbuminemia or normal serum albumin levels.^2^ Ahn et al. also reported that Birmingham vasculitis activity index (BVAS) positively correlated with CRP and negatively with albumin.^3^

C-reactive protein to albumin ratio (CAR), a composite indicator of inflammation and nutritional status, has recently been recognized as an independent prognostic marker in malignancy, Crohn’s disease, critically ill patients and vasculitides.^4–7^ However, data regarding CAR and its relationship with disease activity in AAV patients are scarce. Moon et al. has recently demonstrated that baseline CAR was significantly associated with BVAS and CAR was an independent predictor of all-cause of mortality in patients with newly diagnosed AAV (7). In this study, we aimed to investigate CAR and its relationship with disease activity and damage in prevalent ANCA vasculitis patients.

## MATERIALS AND METHODS

This was a cross-sectional study conducted in 3 centres including 51 prevalent ANCA patients and 42 age- and sex-matched healthy controls, conducted between October 2020 and February 2021. Patients with active infection, malignancy or secondary vasculitis were excluded. Medical history and demographic data were recorded. Blood samples were obtained under fasting conditions. Fasting plasma glucose, kidney function tests, liver function tests, erythrocyte sedimentation rate (ESR) and complete blood counts were measured. C-reactive protein levels were measured using the nephelometric method (Date Behring Siemens, Marburg, Germany) and expressed as milligrams per litre. Creatinine clearance was estimated according to modification of diet in renal disease (MDRD) formula. Birmingham vasculitis activity index (BVAS) version 3 was used to assess disease activity.^8^ Vasculitis damage index was used to provide information on disease damage.^9^ We calculated the CRP to albumin ratio (CAR) (mg/g) by dividing CRP level to albumin level. After data collection ROC curve analysis was performed for the study population and showed that CAR ≥0.98 predicted high BVAS with 70.0% sensitivity and 68.0% specificity (Area under curve: 0.660, CI: 0.482–0.837, p=0.049). AAV patients were further categorized into the two groups according to CAR. Clinical, demographic and laboratory data of the groups were analyzed and compared. The study conforms with the principles outlined in the Declaration of Helsinki. Local ethics committee approved the study and all participants gave written informed consent.

## STATISTICAL ANALYSIS

SPSS (version 22.0; SPSS Inc, Chicago, IL) statistics package was used for statistical analysis. Categorical variables were presented as numbers and percentages and compared with the Chi-square test. Continuous variables were presented as mean ± standard deviation or median (25^th^–75^th^ percentile). Continuous variables with parametric distribution were compared with independent samples t-test, and those without normal distribution were compared with Mann-Whitney U-test. Kolmogorov-Smirnov analysis was performed to determine whether continuous variables were normally distributed. Receiver operating characteristic (ROC) curve analysis was performed to determine the cut off CAR that predicted BVAS ≥5 in AAV patients. Logistic regression analyses were performed to determine independent factors associated with CAR ≥0.98 in patients with vasculitis. Pearson correlation test was used for statistical correlation between CAR, BVAS, VDI, albumin, CRP and ESR. For all statistical analyses, a p-value <0.05 was considered significant.

## RESULTS

CAR was significantly higher in AAV patients than healthy controls [0.95 (0.70–1.34) vs. 0.68 (0.40–0.82); p= 0.006]. Baseline clinical and laboratory data of AAV patients and controls are shown in **Table 1**. The median (25^th^–75^th^) age of the patients was 55 (48–61) years and 25 (49.0%) patients were male. Thirty-two (62.7%) and 19 (37.3%) patients had a diagnosis of GPA and MPA, respectively. Twenty-one patients (41.2%) were positive for p-ANCA and 25 (49.0%) were positive for c-ANCA at diagnosis. The median (25^th^–75^th^) duration of disease was 34 (11–72) months. At the time of the study, current immunosuppressive drug use of the patients were as follows: 32 (62.7%) patients were on oral steroids, 6 (11.8%) patients were on cyclophosphamide, 16 (31.4%) patients were on azathioprine, 2 (3.9%) patients were on mycophenolate mophetil and 15 (29.4%) patients were using rituximab. Cumulative steroid dosage of AAV patients was 6.1 (2.0–11.2) grams The median (25^th^–75^th^) BVAS was 3 (2–5) and VDI was 2 (1–4). Disease activity scores were categorized based on the 75^th^ percentile of BVAS as high (BVAS ≥5) and low (BVAS <5) BVAS. ROC curve analysis showed that CAR ≥0.98 predicted high BVAS with 70.0% sensitivity and 68.0% specificity (Area under curve: 0.660, CI: 0.482–0.837, p=0.049). When patients with CAR ≥0.98 were compared to those without, BVAS [5.0 (3.5–8.0) vs. 2.0 (0–3.25), p< 0.001], BVAS ≥5 [16 (64.0%) vs 4 (15.4%) patients, p=0.001], VDI [4.0 (2.0–4.0) vs. 2.0 (1.0–3.0), p=0.006], leukocyte [8.2 (7.2–12.3) vs. 6.7 (5.6–9.6) x10^3^/μL, p= 0.042], and CRP [5.3 (3.1–13.8) vs. 3.2 (2.6–3.2) mg/L, p=0.001] were significantly higher, whereas albumin [3.8 (3.1–4.3) vs. 4.1 (3.9–4.4) g/dL, p=0.025] and haemoglobin [12.1 (10.4–13.4) vs. 13.0 (12.5–14.2) g/dL, p= 0.008] were significantly lower. The clinical, demographic and laboratory data of both groups are shown in **Table 2** and **Table 3**.

**Table 1.** Baseline clinical and laboratory characteristics of anti-neutrophil cytoplasmic antibody-associated vasculitis patients and controls.

	**Patients (n:51)**	**Controls (n:42)**	**P**
**Age, years**	55 (48–61)	52 (46–60)	0.364
**Male, gender, n (%)**	25 (49.0%)	22 (52.4%)	0.836
**BMI, kg/m^2^**	27.8 (25.5–31.2)	27.0 (24.5–29.5)	0.099
**Smoking, n (%)**	7 (13.7%)	9 (21.4%)	0.411
**Glucose, mg/dL**	92 (84–107)	97 (90–103)	0.518
**HbA1c, %**	5.7 (5.5–6.0)	5.6 (5.3–5.8)	**0.027**
**Creatinine, mg/dL**	1.17 (0.86–2.15)	0.76 (0.61–0.84)	**<0.001**
**GFR, ml/min/1.73 m^2^**	54 (29–80)	103 (97–114)	**<0.001**
**Albumin, g/dL**	4.0 (3.5–4.3)	4.6 (4.4–4.8)	**<0.001**
**Calcium, mg/dL**	9.4 (8.9–9.8)	9.7 (9.4–10.0)	**0.001**
**Phosphorus, mg/dL**	3.6 (3.0–4.2)	3.4 (3.2–3.8)	0.268
**Aspartate transaminase, U/L**	18 (13–21)	19 (17–23)	**0.024**
**Alanine transaminase, U/L**	16 (11–19)	21 (15–27)	**0.002**
**Hemoglobin, g/dL**	12.7 (11.0–13.7)	14.3 (13.2–15.7)	**<0.001**
**Platelet, x10^3^/μL**	284 (247–341)	218 (186–291)	**0.003**
**Leukocyte, x10^3^/mL**	7.7 (6.3–10.9)	6.7 (5.0–8.0)	0.124
**C-reactive protein, mg/L**	3.2 (3.0–5.7)	3.1 (2.0–3.4)	0.078
**Erythrocyte sedimentation rate, mm/h**	31.0 (15.5–39.0)	7.0 (5.0–11.8)	**0.001**
**C-reactive protein to albumin ratio**	0.95 (0.70–1.34)	0.68 (0.40–0.82)	**0.006**
**Duration of disease, months**	34.0 (11.0–72.0)		
**p-ANCA, n (%)**	21 (41.2%)		
**c-ANCA, n (%)**	25 (49.0%)		
**ANCA negative**	5 (9.8%)		
**GPA/MPO, (%)**	62.7/37.3		
**BVAS**	3 (2–5)		
**BVAS ≥5**	20 (39.2%)		
**VDI**	2 (1–4)		
**CAR ≥0.98**	25 (49.0%)		

ANCA: Anti-neutrophil cytoplasmic antibody; BMI: Body mass index; BVAS: Birmingham Vasculitides Activity Index; CAR: C-reactive protein to albumin ratio; c-ANCA: Cytoplasmic anti-neutrophil cytoplasmic antibody; p-ANCA: Perinuclear anti-neutrophil cytoplasmic antibody; VDI: Vasculitis Damage Index. Data presented as median (25^th^–75^th^ percentile).

**Table 2.** Comparison of clinical and demographic data according to C-reactive protein to albumin ratio (CAR).

	**CAR ≥0.98 n:25**	**CAR <0.98 n:26**	**P**
**Glucose, mg/dL**	100.0 (88.5–111.0)	88.5 (81.8–97.2)	0.136
**Creatinine, mg/dL**	1.51 (0.95–2.36)	1.10 (0.84–2.13)	0.136
**GFR, ml/min/1.73m^2^**	45.8 (27.5–73.5)	57.9 (31.2–91.5)	0.294
**Albumin, g/dL**	3.8 (3.1–4.3)	4.1 (3.9–4.4)	**0.025**
**Calcium, mg/dL**	9.3 (8.6–9.8)	9.6 (9.2–9.8)	0.165
**Phosphorus, mg/dL**	3.6 (2.9–4.7)	3.7 (3.1–3.9)	0.664
**Aspartate transaminase, U/L**	18 (13–22)	18 (13–20)	0.910
**Alanine transaminase, U/L**	15 (10–21)	16 (12–20)	0.590
**Hemoglobin, g/dL**	12.1 (10.4–13.4)	13.0 (12.5–14.2)	**0.008**
**Platelet, x10^3^/μL**	306 (249–360)	262 (238–301)	0.129
**Leukocyte, x10^3^/μL**	8.2 (7.2–12.3)	6.7 (5.6–9.6)	**0.042**
**CRP, mg/L**	5.3 (3.1–13.8)	3.2 (2.6–3.2)	**0.001**
**ESR, mm/h**	32.0 (16.3–51.8)	22.5 (12.5–35.5)	0.186

CAR: C-reactive protein to albumin ratio; CRP: C-reactive protein; ESR: Erythrocyte sedimentation rate; GFR: Glomerular filtration rate. Data presented as median (25^th^–75^th^ percentile).

**Table 3.** Comparison of laboratory data according to C-reactive protein to albumin ratio (CAR).

	**CAR ≥0.98 n:25**	**CAR <0.98 n:26**	**P**
**Age, years**	57.0 (51.5–62.0)	50.5 (46.5–59.3)	0.149
**Male, gender, n (%)**	14 (56.0%)	11 (42.3%)	0.406
**BMI, kg/m^2^**	28.1 (24.8–32.2)	27.8 (26.0–30.9)	1.000
**Smoking, n (%)**	4 (16.0%)	3 (11.5%)	0.703
**Duration of disease, months**	18 (5.0–66.0)	36 (18.8–77.5)	0.124
**p-ANCA, n (%)**	8 (32.0%)	13(50.0%)	0.305
**c-ANCA, n (%)**	15 (60.0%)	10(38.5%)	
**ANCA negative**	2 (8.0%)	3(11.5%)	
**Granulomatosis/Microscopic, n**	17/8	15/11	0.565
**BVAS**	5.0 (3.5–8.0)	2.0 (0–3.25)	**<0.001**
**BVAS ≥5**	16 (64.0%)	4 (15.4%)	**0.001**
**VDI**	4.0 (2.0–4.0)	2.0 (1.0–3.0)	**0.006**

ANCA: Anti-neutrophil cytoplasmic antibody, BMI: Body mass index, BVAS: Birmingham Vasculitis Activity Score, c-ANCA: Cytoplasmic anti-neutrophil cytoplasmic antibody, p-ANCA: Perinuclear anti-neutrophil cytoplasmic antibody, VDI: Vasculitis Damage Index. Data presented as median (25^th^–75^th^ percentile).

On correlation analysis, CAR was significantly correlated with BVAS (r: 0.466, p: 0.001). BVAS was also positively correlated with ESR (r: 0.464, p: 0.001) and negatively correlated with albumin (r: −0.614, p= <0.001). However, there was no correlation between BVAS and CRP (r: 0.251, p= 0.076). On the other hand, cumulative steroid dosage was negatively correlated with BVAS (r: −0.518, p< 0.001), there was no correlation with VDI (r: 0.006, p= 0.969) and CAR (r: −0.068, p= 0.642).

Multivariate analysis revealed that haemoglobin [OR (95% CI): 0.475 (0.255–0.885), p= 0.019] and BVAS [OR (95% CI): 1.313 (1.003–1.719), p= 0.047] were independent factors associated with CAR ≥0.98 in patients with AAV (**Table 4**).

**Table 4.** Independent factors associated with CAR ≥0.98 in patients with AAV.

	**OR (95% CI)**	**P**
**Gender**	0.190 (0.030–1.199)	0.077
**Age**	1.000 (0.929–1.075)	0.989
**Haemoglobin**	0.475 (0.255–0.885)	**0.019**
**BVAS**	1.313 (1.003–1.719)	**0.047**
**VDI**	1.752 (0.965–3.180)	0.065

BVAS: Birmingham vasculitis activity score; OR: Odd’s ratio; VDI: Vasculitis damage index.

## DISCUSSION

In the present study, we showed that CAR is associated with high disease activity in AAV patients. CRP and serum albumin are well known acute phase reactants that have been used as critical inflammatory biomarkers for predicting morbidity and mortality in various diseases.^10,11^ During inflammation, CRP levels increase whereas albumin levels decrease. Albumin is also an indicator of nutritional status. Ahn et al. demonstrated that the controlling nutritional status score, which was developed to detect under-nutrition in patients, was associated with BVAS and all-cause mortality in 196 newly diagnosed AAV patients.^12^ In our study, patients with CAR ≥0.98 also had higher BVAS.

The CAR combines systemic inflammation and nutritional status and has become an area of interest for several inflammatory, infectious and malignant disorders.^6,7^ It may be more valuable as a prognostic marker for patient outcomes and subsequent studies have shown that CAR is more consistent with prognosis than CRP or albumin alone.^13,14^

In the only other study evaluating CAR in AAV patients, Moon et al. showed that baseline CAR was significantly associated with BVAS at diagnosis and CAR was an independent predictor of all-cause of mortality.^7^ In our study, the patient population was different in the sense that we included ANCA patients with a median disease duration of 34 months. Therefore, we have shown that CAR is also associated with BVAS in prevalent patients and thus can be used during follow-up. In a retrospective study on 160 patients with rheumatoid arthritis and 159 healthy controls, CAR was found to be higher than controls and positively correlated with disease activity.^15^ Similarly, Sunar et al. showed that CAR was correlated with the disease activity score in 121 rheumatoid arthritis patients.^16^ In another study by Akkececi et al., the authors showed that CAR was associated with disease activity and decreased in remission in patients with Takayasu arteritis.^17^ In line with the aforementioned studies, we also found that CAR was correlated with disease activity as assessed by BVAS in ANCA vasculitis patients.

Currently the cut-off serum CAR level to predict disease activity in AAV patients is unknown. In the study by Moon et al., patients with CAR ≥10.35 and diabetes exhibited a higher frequency of all-cause mortality than those without.^7^ CAR has been shown to be an independent predictor of all-cause mortality in many disorders such as cancers, inflammatory diseases and septic conditions.^6,18,19^ However, various cut-off levels have been proposed in different studies. A cut-off level greater than 0.189 and 0.38 have been shown to be associated with overall survival in patients with metastatic nasopharyngeal and gastric cancer, respectively.^5,20^ In postoperative patients admitted to the intensive care unit, the probability of death within 30 days or 1 year after admission was higher in patients with CAR ≥1.75 or ≥1.58), respectively.^21^ In another study, the cut-off value of CAR in predicting the 90-day mortality for patients with acute ischemic stroke was 0.50.^22^ Furthermore, different cut-off levels have been reported to predict disease activity in patients with inflammatory bowel disease, ranging from 0.18 to 0.60.^23,24^ Data regarding CAR in AAV patients is scarce and cut-off levels are currently unknown, as mentioned above.

Ongoing disease activity and persistent chronic inflammation are associated with poor patient outcomes. Thus, early recognition and timely treatment may limit disease specific morbidity and mortality. In AAV patients, BVAS is one of the main tools to assess disease activity. Flossmann et al. showed that BVAS ≥2 was associated with all-cause mortality in 535 AAV patients over a follow-up time of 5.2 years.^25^ In the present study, we used CAR in addition to BVAS to assess disease activity in AAV patients. We categorized disease activity scores based on the 75^th^ percentile of BVAS as high (BVAS ≥5) and low (BVAS<5) disease activity. CAR was correlated with high disease activity on multivariate analysis. Since CAR is easily calculated from the routine clinical laboratory tests, we propose that it can be used as a simple, feasible and inexpensive method to monitor disease activity and possibly predict prognosis. Larger studies with longer follow-up time are needed to determine whether CAR predicts patient associated outcomes.

Over time, patients with AAV may develop chronic organ damage due to vasculitis itself or the treatments used. The VDI was developed as a tool to distinguish chronic damage from active inflammation or persistent disease. A VDI score >4 was associated with mortality in vasculitis patients.^26^ Another study showed that a baseline VDI score >1 was strongly predictive of mortality in patients with GPA.^27^ In our study median VDI was 4 and was significantly higher in patients with CAR ≥0.98.

The present study has several limitations. The relatively small sample size of the study and cross-sectional nature are the main limitations. However, we don’t have any data of patients’ baseline CAR and BVAS status. It would be interesting to follow-up patients to determine whether CAR predicts patient related morbidity and mortality. We also did not evaluate the nutritional status of our patients. Inflammation markers are essential for predicting disease related morbidity and mortality in AAV patients. In the present study, we found that CAR, a novel inflammation marker, was an independent predictor of disease activity in AAV patients. Close monitoring, early recognition and timely treatment of disease activity may limit patient morbidity and mortality. Studies with a large sample size and long-term follow up are needed to determine whether CAR predicts patient outcomes.

## Data Availability

All relevant data were presented in the manuscript.
